# A Bayesian mixed modeling approach for estimating heritability

**DOI:** 10.1186/s12919-018-0131-z

**Published:** 2018-09-17

**Authors:** Haakon E. Nustad, Christian M. Page, Andrew H. Reiner, Manuela Zucknick, Marissa LeBlanc

**Affiliations:** 1 Department of Medical Genetics, Oslo University Hospital, Kirkeveien 166, 0450 Oslo, Norway; 2 Department of Medical Genetics, University of Oslo, Klaus Torgårds vei 3, 0372 Oslo, Norway; 3 PharmaTox Strategic Research Initiative, University of Oslo, Sem Sælands vei 3, 0371 Oslo, Norway; 4 Oslo Centre for Biostatistics and Epidemiology, Oslo University Hospital, Klaus Torgårds vei 3, 0372 Oslo, Norway; 5 Department of Non-Communicable Disease, Norwegian Institute of Public Health, Marcus Thranes gate 6, 0473 Oslo, Norway; 6 Oslo Centre for Biostatistics and Epidemiology, University of Oslo, Sognsvannsveien 9, 0372 Oslo, Norway

## Abstract

**Background:**

A Bayesian mixed model approach using integrated nested Laplace approximations (INLA) allows us to construct flexible models that can account for pedigree structure. Using these models, we estimate genome-wide patterns of DNA methylation heritability (*h*^*2*^), which are currently not well understood, as well as *h*^*2*^ of blood lipid measurements.

**Methods:**

We included individuals from the Genetics of Lipid Lowering Drugs and Diet Network (GOLDN) study with Infinium 450 K cytosine-phosphate-guanine (CpG) methylation and blood lipid data pre- and posttreatment with fenofibrate in families with up to three-generation pedigrees. For genome-wide patterns, we constructed 1 model per CpG with methylation as the response variable, with a random effect to model kinship, and age and gender as fixed effects.

**Results:**

In total, 425,791 CpG sites pre-, but only 199,027 CpG sites posttreatment were found to have nonzero heritability. Across these CpG sites, the distributions of *h*^*2*^ estimates are similar in pre- and posttreatment (*pre:* median = 0.31, interquartile range [IQR] = 0.16; *post:* median = 0.34, IQR = 0.20). Blood lipid *h*^*2*^ estimates were similar pre- and posttreatment with overlapping 95% credibility intervals. Heritability was nonzero for treatment effect, that is, the difference between pre- and posttreatment blood lipids. Estimates for triglycerides *h*^*2*^ are 0.48 (pre), 0.42 (post), and 0.21 (difference); likewise for high-density lipoprotein cholesterol *h*^*2*^ the estimates are 0.61, 0.68, and 0.10.

**Conclusions:**

We show that with INLA, a fully Bayesian approach to estimate DNA methylation *h*^*2*^ is possible on a genome-wide scale. This provides uncertainty assessment of the estimates, and allows us to perform model selection via deviance information criterion (DIC) to identify CpGs with strong evidence for nonzero heritability.

## Background

Narrow-sense heritability (*h*^*2*^), traditionally estimated using twins or other constrained family relationships, can also be estimated in wider pedigrees using a linear mixed-model approach [1, 2]. This approach is well-established for traits having moderate to high *h*^*2*^ [2, 3]. What is less clear is how well these models perform for traits with low *h*^*2*^, which is the case for some proportion of cytosine-phosphate-guanine (CpG) methylation sites genome wide. This is of particular interest for *h*^*2*^ because it is a ratio of variance components, that is, the proportion of phenotypic variance explained by additive genetic variance. Compared to estimates of the mean, estimates of variance tend to have large uncertainty.

DNA methylation (DNAm) is an epigenetic mark that is implicated in many heritable diseases and traits. DNAm patterns are influenced by the environment, change over lifetime, and exhibit mitotic heritability [4]. There are multiple reports of DNAm *h*^*2*^ with CpG-specific estimates ranging from 0 to 1, and with genome-wide mean or median estimates ranging from approximately 15 to 30% [5–7].

Any point estimates of *h*^*2*^ should ideally be accompanied by an assessment of the uncertainty. A Bayesian mixed model with inference by integrated nested Laplace approximations (INLA) [2, 8] presents an improvement on the traditionally used simple, balanced designs (eg, parent–offspring or twin-based correlations) as it can include the full pedigree structure. Additionally, full posterior inference provides uncertainty for the estimated *h*^*2*^, which is not obtainable using frequentist methods, and allows an assessment of whether the true *h*^*2*^ is nonzero via comparison of models with and without a genetic component.

In this article, we use three-generation pedigrees from the Genetics of Lipid Lowering Drug and Diet Network (GOLDN) [9] to estimate *h*^*2*^ in a fully Bayesian framework. Blood lipid and DNAm measurements were available pre- and posttreatment with a lipid-lowering drug (fenofibrate). We first investigate continuous blood lipid traits with known moderate to high *h*^*2*^ and compare the Bayesian estimates with previously published frequentist estimates [10]. Second, we estimate the genome-wide distribution of DNAm *h*^*2*^.

The Bayesian mixed-model approach provides posterior distributions and therefore uncertainty for *h*^*2*^, the additive genetic variance, and the environmental variance. We further investigate if it is reasonable to estimate *h*^*2*^ for each CpG site given the data by performing model selection, to assess if the genetic component should be included at all. When the additive genetic variance is truly zero, a linear mixed model including the family structure will misspecify the model, making any resultant *h*^*2*^ estimates untrustworthy. When *h*^*2*^ is truly nonzero but smaller than the minimum *h*^*2*^ detectable in a given data set (ie, when the study is underpowered), we would expect extremely large standard errors for the *h*^*2*^ estimates.

In this article, our aims are to estimate *h*^*2*^ for (a) blood lipid traits and (b) genome-wide DNAm using a Bayesian approach, including a model selection step to identify CpG sites with strong evidence for nonzero heritability. We highlight the importance of the model selection step and of quantifying the uncertainty in *h*^*2*^ estimates.

## Methods

### Data

Individuals in the GOLDN study having DNAm and blood lipid measurements, as well as information on gender and age, were included in the analysis [11]. Genome-wide DNAm was measured using the 450 K Infinium array at 463,995 CpG sites and was available for 995 and 530 individuals at pre- and postfenofibrate treatment, respectively. From the original 463,995 sites available, we removed a list of known single nucleotide polymorphism–related CpG sites. Blood lipid levels were available both pre- and posttreatment for 818 and 861 individuals for triglycerides (TGs) and high-density lipoproteins (HDLs), respectively. In our analyses, we use log-transformed TG and HDL (averaged over 2 measurements taken 1 day apart), and simply refer to these transformed average values as TG and HDL from here on.

Standard quality control inspection of the DNAm data showed systematic differences with respect to probe-type chemistry. We therefore normalized the data with respect to the probe type using beta-mixture quantile normalization using the *R* package *wateRmelon* [12]. We calculated the M values for each probe and used these in the analysis.

### Statistical model

We estimate *h*^*2*^ in a Bayesian framework using the INLA package in Rue et al. [8]. Using the following model, we investigate *h*^*2*^ for TG, HDL, and DNAm in the GOLDN study pre- and posttreatment:

$$ {y}_i\mid {\eta}_i,{\sigma}_e^2\sim N\left({\eta}_i,{\sigma}_e^2\right)\forall i=1,\dots, n\ \mathrm{with}\ {\eta}_i={\beta}_0+{\boldsymbol{x}}_i^T\boldsymbol{\beta} +{u}_i $$

1 $$ \boldsymbol{u}\mid {\sigma}_g^2\sim N\left(\mathbf{0},2{\sigma}_g^2\boldsymbol{K}\right)\ \mathrm{with}\ \boldsymbol{u}=\left({u}_1,\dots, {u}_n\right) $$

where ***y*** = (*y*_1_, …, *y*_*n*_) is the outcome vector, that is, DNAm for a given CpG, TG, or HDL, for *n* subjects. $N\left({\eta}_i,{\sigma}_e^2\right)$ is the likelihood function for subject *i*, where *η*_*i*_ is the linear predictor defining the latent variables of interest and ${\sigma}_e^2$ denotes the residual unexplained variance in the model. Fixed effects are the intercept *β*_0_ and ***β*** = (*β*_1_, *β*_2_) corresponding to the effects of age (x_1i_) and gender (x_2i_). Family structure was modeled with ***u*** as a random effect, with distribution$N\left(\mathbf{0},2{\sigma}_g^2\boldsymbol{K}\right)$, where ***K*** is the kinship matrix calculated based on pedigree information using the *{kinship2}* package in R [13], and ${\sigma}_g^2$ is the additive genetic variance. We assume default noninformative priors for all hyperparameters for the genome-wide investigation of *h*^*2*^ ($\mathrm{in}\ \mathrm{particular}\ {\sigma}_{\ast}^{-1}\sim Gamma\left(\mathrm{1,0.00005}\right)$), but other priors were also investigated. From this model,${h}^2=\frac{\sigma_g^2}{\sigma_g^2+{\sigma}_e^2}$.

With the deviance information criterion (DIC) [14, 15], we can investigate the fit of a model to the data and do model selection. We can compare the DIC of the 2 latent models:

2 $$ {\eta}_i={\beta}_0+{\boldsymbol{x}}_i^T\boldsymbol{\beta} $$

3 $$ {\eta}_i={\beta}_0+{\boldsymbol{x}}_i^T\boldsymbol{\beta} +{u}_i $$

and use the recommended ΔDIC = DIC_(2)_ − DIC_(3)_ > 10 to choose if we should include the genetic component (*u*_*i*_) or not.

As a result of the setup of INLA, we can only obtain point estimates of *h*^*2*^ with this model formulation, but not the marginal posterior distribution. If this is of interest, the following equivalent model formulation must be used instead [2]. The nuisance parameter ${\sigma}_0^2$ is introduced to ensure that ${\sigma}_e^2$ remains identifiable. It is fixed at a small value, so that it does not absorb much of the variation in the data and thus does not interfere with ${\sigma}_g^2$ or ${\sigma}_e^2$. We used ${\sigma}_0^2=4.54\mathrm{e}-5$, which corresponds to log(σ^2^_0_) = − 10 and was suggested in an earlier publication [2].

$$ {y}_i\mid {\eta}_i,{\sigma}_e^2\sim N\left({\eta}_i,{\sigma}_0^2\right)\forall i=1,\dots, n\ \mathrm{with}\ {\eta}_i={\beta}_0+{\boldsymbol{x}}_i^T\boldsymbol{\beta} +{u}_i+{\varepsilon}_i $$

$$ \boldsymbol{u}\mid {\sigma}_g^2\sim N\left(\mathbf{0},2{\sigma}_g^2\boldsymbol{K}\right)\mathrm{with}\ \boldsymbol{u}=\left({u}_1,\dots, {u}_n\right) $$

$$ \boldsymbol{\varepsilon} \mid {\sigma}_e^2\sim N\left(\mathbf{0},{\sigma}_e^2\boldsymbol{I}\right)\ \mathrm{with}\ \boldsymbol{\varepsilon} =\left({\varepsilon}_1,\dots, {\varepsilon}_n\right) $$

## Results

**Fig. 1:**
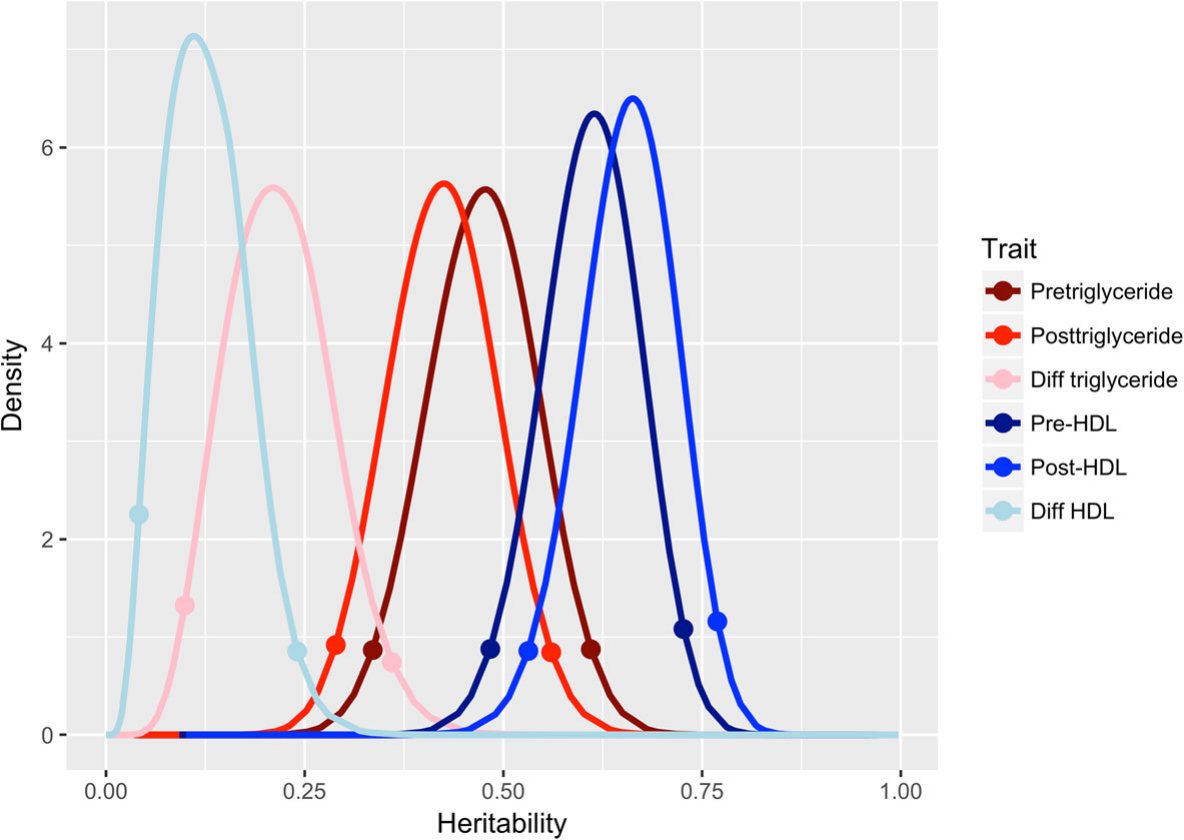
Heritability *(h*^*2*^) estimates for high-density lipoprotein (HDL) and triglycerides (TG). Posterior *h*^*2*^ distribution for HDL and TG pre- and posttreatment, as well as for the change from pre- to posttreatment (“Diff”). The points indicate the 95% credibility region for each *h*^*2*^ estimate

Figure 1 shows the marginal posterior distribution of *h*^*2*^ for HDL and TG levels pre- and posttreatment, as well as for the change (post–pre). The change in blood lipid levels can be interpreted as the response to treatment. The model selection step supported the inclusion of *u*_*i*_ in the model and thus estimation of *h*^*2*^ for each case. The mode estimates of *h*^*2*^ for TG are 0.48, 0.42, and 0.21 for baseline, posttreatment, and change, respectively. Likewise, the mode estimates for *h*^*2*^ for HDL are 0.61, 0.68, and 0.10. Based on point estimates alone, HDL appears to be more heritable than TG but their 95% credible intervals overlap. TG is more heritable pretreatment than posttreatment, but the opposite is true for HDL. However, the 95% credibility intervals for *h*^*2*^ pre- and posttreatment overlap each other substantially for both traits.

**Fig. 2:**
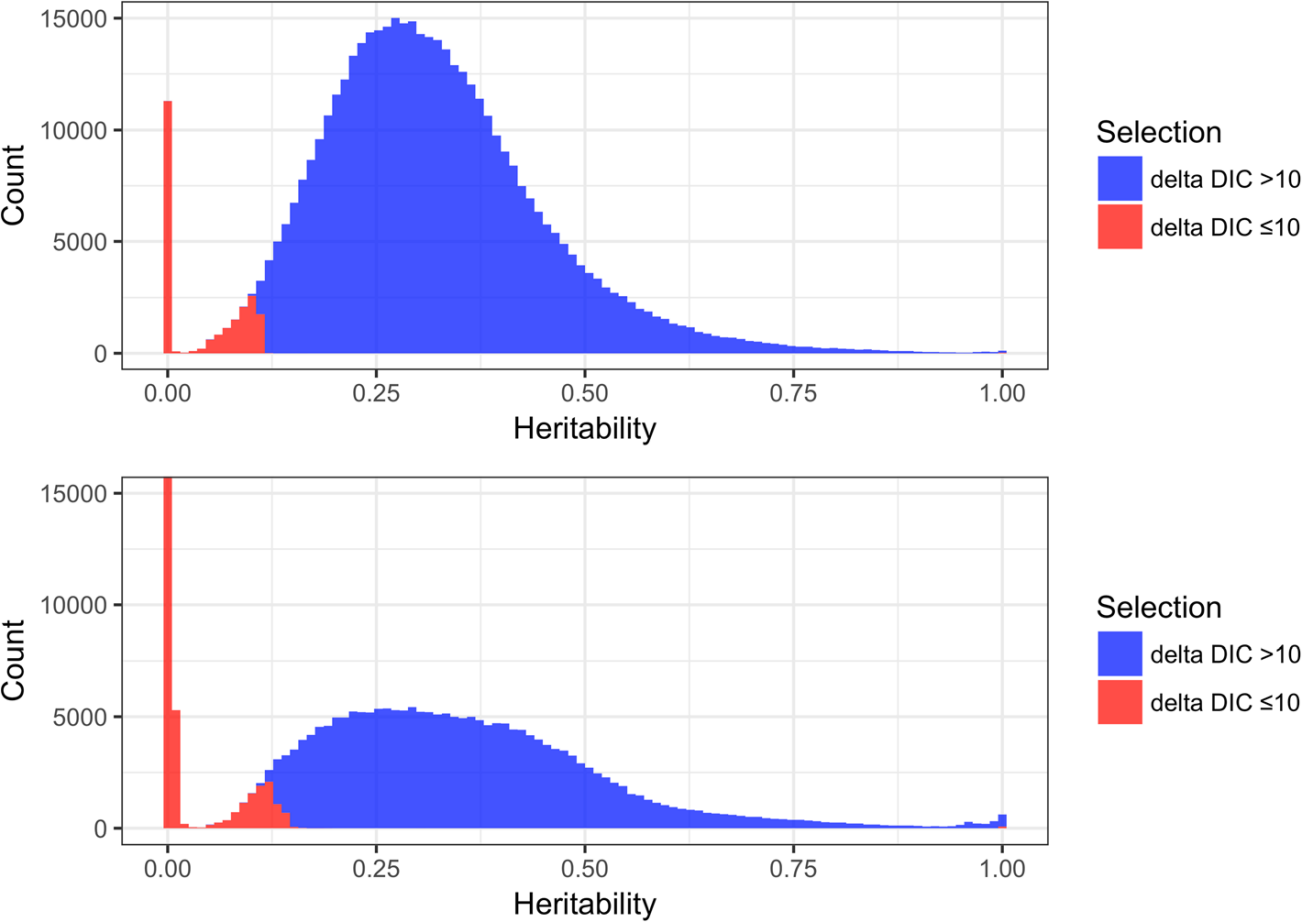
Genome-wide heritability (*h*^*2*^) estimates for DNA CpG methylation (DNAm). Histogram of point estimates for DNAm *h*^2^ genome wide. The top panel shows the *h*^*2*^ estimates pretreatment and the lower panel shows the *h*^*2*^ estimates posttreatment. The blue color is for CpG sites with strong evidence for heritability (“delta DIC >10”) and the red color is for the CpG sites with no evidence for heritability given the data (“delta DIC ≤10”), based on model selection comparing the models with and without pedigree information [eqs. (2) and (3)]. The two colors are stacked on top, so that both colors are visible on each bar. The actual height of the zero-stack bar for the posttreatment is 238,165 CpG sites

In Fig. 2, the histogram of point estimates of *h*^*2*^ of DNAm for 448,040 CpG sites is displayed, for both pre- and posttreatment. Model selection indicates if there is evidence for nonzero *h*^*2*^.

For pretreatment, we detect nonzero *h*^*2*^ for 425,791 of 448,040 CpG sites (95.0%). For the CpG sites with strong evidence for nonzero *h*^2^, the mean, median, and interquartile range (IQR) are 0.33, 0.31, and 0.16. In contrast, for posttreatment we detect nonzero *h*^*2*^ for 199,027 of 448,040 CpG sites (44.4%) with mean, median, and IQR equal to 0.36, 0.34, and 0.20. Both cases show evidence of zero inflation and a right skewed distribution of DNAm *h*^*2*^. Indirectly, we see that GOLDN is underpowered for detecting *h*^*2*^ of DNAm less than approximately 10 to 15%.

**Fig. 3:**
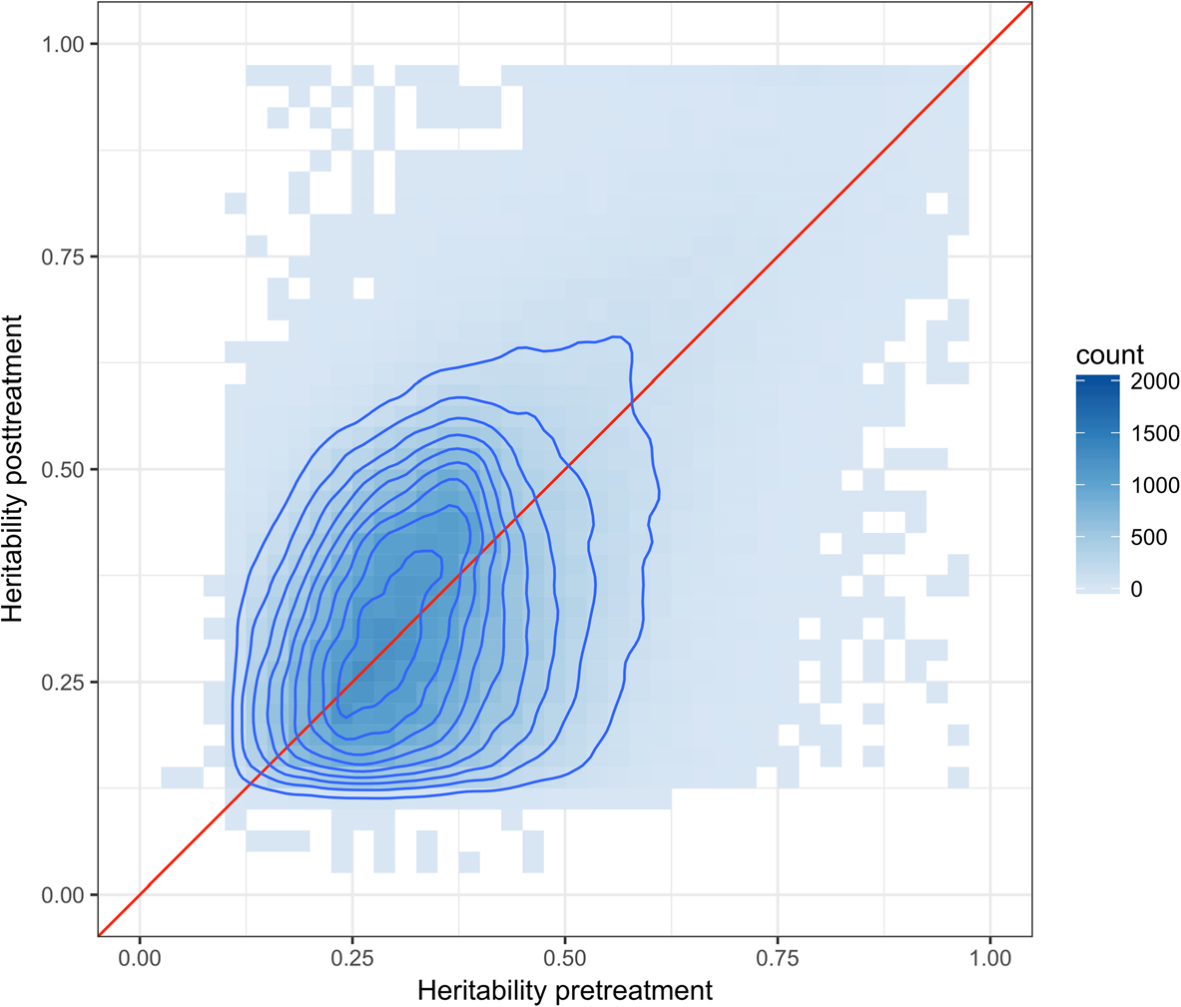
Contour plot of correlation of DNA CpG methylation heritability (*h*^*2*^) pre- and posttreatment. The plot is based on the 194,741 CpG sites that have evidence for nonzero heritability in both pre- and posttreatment methylation measurements

Figure 2 clearly shows a striking difference in the distribution of genome-wide *h*^*2*^ estimates between pre- and posttreatment. Figure 3 shows the direct pairwise comparison of the two *h*^*2*^ estimates for DNAm across those CpG sites with strong evidence for nonzero *h*^*2*^. For these CpG sites, we observe a moderate Pearson correlation between the baseline and posttreatment heritability estimates of 0.53.

## Discussion and conclusions

In this article, we use a Bayesian approach to estimate *h*^*2*^ for blood lipid levels and DNAm in extended pedigrees, and show that it is feasible to do genome-wide Bayesian estimation of *h*^*2*^.

For blood lipids, our pretreatment point estimates for *h*^*2*^ using the Bayesian approach (*h*^*2*^_TG_ = 0.48 and *h*^*2*^_HDL_ = 0.61) are comparable to previously reported estimates using other methods [16]. Notably, the 95% credibility intervals are wide (see Fig. 1). Interestingly, the response to treatment (change in blood lipid levels from pre- to posttreatment; see Fig. 1) also appears to be heritable. This may have important implications for personalized medicine, especially if one were able to identify explanatory variables (genetic or environmental) that predict the individual genetic value (*u*_*i*_).

The genome-wide distribution of DNAm *h*^*2*^ (as ascertained by the Illumina Infinium 450 K chip) appears to follow a 2-group mixture model with a *h*^*2*^ = 0 component and a nonzero component following a smooth, unimodal right skewed distribution. The mixture proportion is strikingly different in the pre- and posttreatment (see Fig. 2). It is unlikely that treatment with fenofibrate could cause substantial genome-wide changes in DNAm *h*^*2*^. It is much more likely that technical differences between the DNAm time points explain the difference [17].

The *h*^*2*^ estimates we present in this article are generally similar to earlier genome-wide results in different populations [5, 7]. These previously published studies also show zero inflation, although the reported proportions vary substantially, from 5 to 17%. All show right-skewed distributions, but with slightly different modality. We report the median, *h*^*2*^_pre_ = 0.31, *h*^*2*^_post_ = 0.34, in the GOLDN study only for CpG sites with strong evidence for nonzero *h*^*2*^ (ie, ΔDIC > 10). This is higher than previously reported, although this is to be expected if previous reports included the zero component in the calculation of the median. With the zero component included, our median estimates decrease to *h*^*2*^_pre_ = 0.30, *h*^*2*^_post_ = 0.0036.

A Bayesian model holds some advantages over a frequentist model when estimating *h*^*2*^. The most notable difference is that a Bayesian model can estimate the full posterior distribution of the parameters in question. This includes the random effect variance parameters, which allows more direct assessment of the uncertainty of the random-effect estimates—and thus of the *h*^*2*^ estimates—than could be achieved with classical mixed-effects models. For example, by comparing posterior credibility intervals for *h*^*2*^ pre- versus posttreatment, we can determine if the *h*^*2*^ changes are a result of treatment (see Fig. 1). In addition, model selection can be performed in a principled way by DIC or Bayes factors to assess if the data provide sufficient evidence for nonzero *h*^*2*^. When the *h*^*2*^ is truly zero, the genetic values *u*_*i*_ should not be included in the model, and therefore a model without the genetic values should be favored. Indeed, DIC-based model selection favored the model without random effect in the vast proportion of CpG sites for which *h*^*2*^ was estimated to be close to zero.

An important limitation in our study is the lack of information on shared households to estimate the effect of shared environment. This likely results in an overestimation of *h*^*2*^. The CpG sites on the 450 K array are a nonrandom selection of CpG sites with a bias toward CpG islands that often have regulatory function. This is important to consider, as an oversampling of biologically important CpG sites could result in a biased estimation for nonspecific genome-wide DNAm *h*^*2*^.

In this article, we use a Bayesian approach to estimate *h*^*2*^ in extended pedigrees and show the importance of using model selection to determine if there is strong evidence of nonzero *h*^*2*^ given the data. Future work should focus on simulation studies comparing the frequentist and Bayesian approaches, with particular focus on traits having low *h*^*2.*^.
